# Supplementary material for: From ice cores to dinosaurs: physical collections managers’ research data curation perceptions and behaviors
Source: PLoS One. 2025 Jun 27;20(6):e0317070. doi: 10.1371/journal.pone.0317070 (PMC12204691; doi:10.1371/journal.pone.0317070)
Supplement: Attachment — Submitted filename: ResponsetoReviewers.pdf [file pone.0317070.s002.pdf]

Dear academic editors and reviewers of *PLOS One*,

The manuscript, *From Ice Cores to Dinosaurs: Physical Collections Managers' Research Data Curation Perceptions and Behaviors*, has been greatly strengthened by addressing all your suggested changes to the manuscript. The following list summarizes the changes to the manuscript.

- The abstract sentence Reviewer #2 found confusing was edited to say "managers note that these physical collections and derived data reuse require more investments" for clarity.
- We have cut all mention from the acknowledgments section.
- The statement was added to the Methods section "The University of Tennessee Institutional Review Board approved this study November 9, 2022 (UTK IRB-22-07183-XP)."
- The method also includes a full synopsis of the method including two additional citations to prior work.
- Additional thoughts were added to sufficient metadata for reuse section based on Reviewer #1 suggestion.
- Discussion section now includes more about the benefits and limitations of the DCP method.

Thank you for your consideration,

Wade Bishop
Professor
Director of Graduate Studies
Research Security Ambassador
Research Data Management Coordinator
School of Information Sciences
University of Tennessee
1345 Circle Park Dr. Room 454
Communications Bldg.
Knoxville, TN 37996
wade.bishop@utk.edu
865-974-2775
