# Supplementary material for: From ice cores to dinosaurs: physical collections managers’ research data curation perceptions and behaviors
Source: PLoS One. 2025 Jun 27;20(6):e0317070. doi: 10.1371/journal.pone.0317070 (PMC12204691; doi:10.1371/journal.pone.0317070)
Supplement: Attachment — Submitted filename: Article review.docx [file pone.0317070.s001.docx]

**Article review**

This article investigates data management practices connected to a group of interdisciplinary physical collections to better understand how cyberinfrastructure tools may promote data reuse. They key insights cover the importance of metadata, the need for greater standardization of data, and a shortage of resources needed to upskill workers or hire staff with cyberinfrastructure skills.

The article would appeal to a moderately broad readership within the fields of museum and library studies. The study is well-designed and emphasizes the integration of insights from a diverse group of collection managers across the natural, physical, and social sciences and humanities. Yet the study also has limitations, which are not adequately addressed beyond a brief mention in the discussion on p.20. The qualitative insights derived from open-ended questioning of physical collection managers are interesting, but also subjective. This makes it somewhat challenging for a reader to integrate findings from this study into their own data management practices. A straightforward discussion of the benefits and limitations of the study design would go a long way toward addressing this issue.

The research design relies on the data curation profiling (DCP) method to guide the focus group questioning. The method is not fully explained in this article, although the questions asked to participants are listed, which is helpful. The article does reference other published work about the method (p.4), however a synopsis would be useful.

The article addresses a significant problem, distilling “the most important reuse facets that could inform the design of the cyberinfrastructure tools and services to improve reusability of all physical collections and resulting data” (p.2). It is increasingly important to curate data in ways that adhere to FAIR and CARE principles – and this article collects data that inform these forward-looking practices. The article also does a good job of referencing previous related research, including RDM (Research Data Management), responsible conduct in research, and the pressure for reproducibility in research (p.3).

I applaud the authors for making their data used in this study fully available without restriction, which is a testament to their commitment to open data and data reuse.

Overall, the paper is written in clear and correct English. There is one sentence I found confusing in the abstract (p.1), that I would recommend revising for clarity. The issue is the distinction between physical collections and interdisciplinary physical collections. The sentence reads, “Results indicated that physical collections attempt to use universal metadata and data storage standards to increase discoverability, but interdisciplinary physical collections and derived data reuse require more investments to increase reusability of these invaluable items.”

Overall, this is a well-researched and well-written paper that summarizes the results of important research. My main suggestion that would expand its relevance to a broader readership within museums and libraries is the addition of a discussion about the benefits and limitations of the study/RCP method.
